# Crystal structure of (*E*)-4-{[2-(2,4-di­nitro­phen­yl)hydrazin-1-yl­idene]meth­yl}-3-methyl-1-phenyl-5-(1*H*-pyrrol-1-yl)-1*H*-pyrazole

**DOI:** 10.1107/S1600536814024039

**Published:** 2014-11-08

**Authors:** Joel T. Mague, Shaaban K. Mohamed, Mehmet Akkurt, Hussein M. S. El-Kashef, Mustafa R. Albayati

**Affiliations:** a Department of Chemistry, Tulane University, New Orleans, LA 70118, USA; b Chemistry and Environmental Division, Manchester Metropolitan University, Manchester M1 5GD, England; c Chemistry Department, Faculty of Science, Minia University, 61519 El-Minia, Egypt; d Department of Physics, Faculty of Sciences, Erciyes University, 38039 Kayseri, Turkey; e Department of Chemistry, Faculty of Science, Assiut University, 71515 Assiut, Egypt; f Department of Chemistry, College of Science, Kirkuk University, Kirkuk, Iraq

**Keywords:** crystal structure, pyrazole, azo­pyrazole, hydrogen bonding

## Abstract

The title compound, C_21_H_17_N_7_O_4_, is in an ‘extended’ conformation aided by an intra­molecular N—H⋯O hydrogen bond. The pyrazole ring makes dihedral angles of 29.17 (6), 65.47 (4) and 9.91 (7)°, respectively, with the phenyl, pyrrole and benzene rings. In the crystal, mol­ecules are connected by pairs of N—H⋯O and C—H⋯O hydrogen bonds, forming inversion dimers which associate into ribbons running along the *b* axis through complementary C—H⋯O inter­actions.

## Related literature   

For the use of pyrazole compounds as building blocks of various heterocyclic compounds, see: Abramov *et al.* (2001 ▶); Quiroga *et al.* (2001 ▶); Wu *et al.* (2006 ▶); El-Emary (2006 ▶); Rangnekar & Dhamnaskar (1988 ▶). For the bioactivity of pyrazole-containing compounds, see: Mashevskaya *et al.* (2001 ▶); Janus *et al.* (1999 ▶); Park *et al.* (2005 ▶); Bouabdallah *et al.* (2006 ▶); Yıldırım *et al.* (2005 ▶); Bailey *et al.* (1985 ▶); Chu & Cutler (1986 ▶). For industrial applications of azo­pyrazole derivatives, see: Karci & Demircah (2006 ▶); Vicentini *et al.* (1998 ▶).
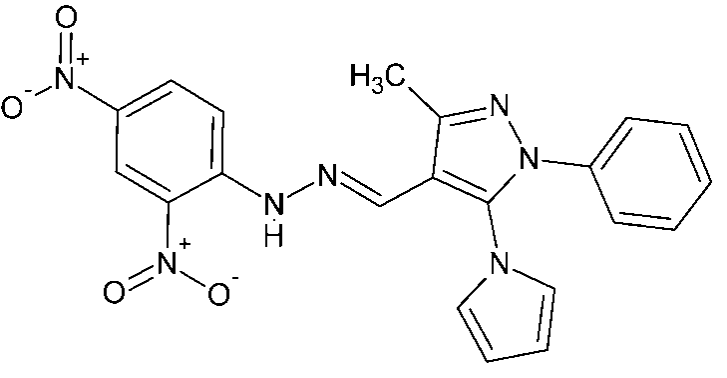


## Experimental   

### Crystal data  

C_21_H_17_N_7_O_4_
*M* *_r_* = 431.42
Monoclinic, 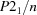

*a* = 5.7955 (1) Å
*b* = 15.4472 (4) Å
*c* = 21.9289 (5) Å
β = 93.831 (1)°
*V* = 1958.78 (8) Å^3^
*Z* = 4
Cu *K*α radiation
μ = 0.88 mm^−1^
*T* = 150 K
0.17 × 0.10 × 0.09 mm

### Data collection  

Bruker D8 VENTURE PHOTON 100 CMOS diffractometer
Absorption correction: numerical (*SADABS*; Bruker, 2014 ▶) *T*_min_ = 0.89, *T*_max_ = 0.93
30722 measured reflections
3854 independent reflections
3425 reflections with *I* > 2σ(*I*)
*R*_int_ = 0.034

### Refinement  

*R*[*F* ^2^ > 2σ(*F* ^2^)] = 0.033
*wR*(*F* ^2^) = 0.089
*S* = 1.03
3854 reflections
290 parameters
H-atom parameters constrained
Δρ_max_ = 0.20 e Å^−3^
Δρ_min_ = −0.27 e Å^−3^

### 

Data collection: *APEX2* (Bruker, 2014 ▶); cell refinement: *SAINT* (Bruker, 2014 ▶); data reduction: *SAINT*; program(s) used to solve structure: *SHELXT* (Sheldrick, 2008 ▶); program(s) used to refine structure: *SHELXL2014* (Sheldrick, 2008 ▶); molecular graphics: *DIAMOND* (Brandenburg & Putz, 2012 ▶); software used to prepare material for publication: *SHELXTL* (Sheldrick, 2008 ▶).

## Supplementary Material

Crystal structure: contains datablock(s) global, I. DOI: 10.1107/S1600536814024039/is5380sup1.cif

Structure factors: contains datablock(s) I. DOI: 10.1107/S1600536814024039/is5380Isup2.hkl

Supporting information file. DOI: 10.1107/S1600536814024039/is5380Isup3.cml

**Fig. 1.**
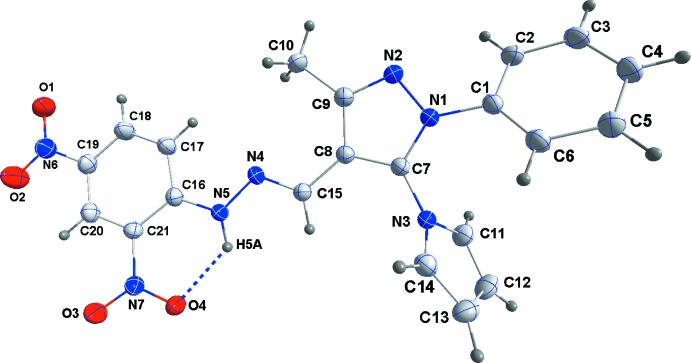
Numbering scheme for the title molecule. Ellipsoids are drawn at the 50% probability level.

**Fig. 2.**
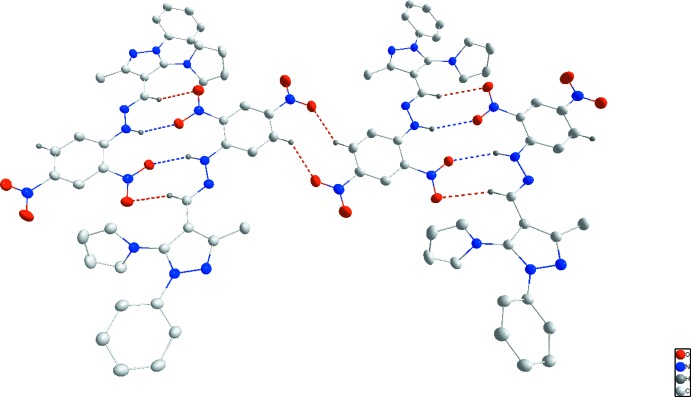
Packing diagram looking down the c axis showing two chains formed by complementary N—H···O and C—H···O interactions and their association through additional C—H···O interactions.

**Fig. 3.**
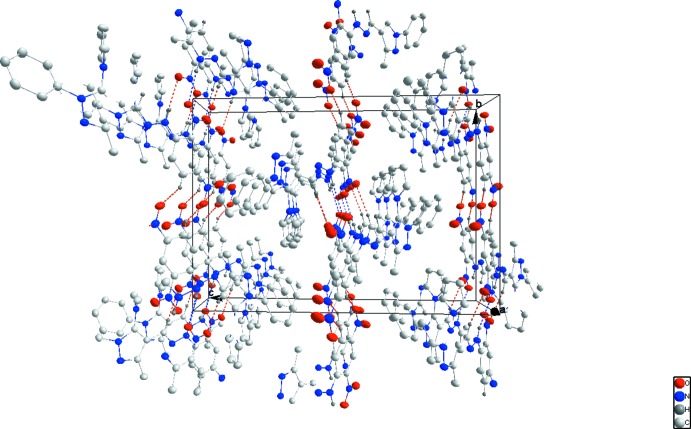
Packing diagram viewed down the a axis showing an edge view of several ribbons.

. DOI: 10.1107/S1600536814024039/is5380fig1.tif
Numbering scheme for the title mol­ecule. Ellipsoids are drawn at the 50% probability level.

c . DOI: 10.1107/S1600536814024039/is5380fig2.tif
Packing diagram looking down the *c* axis showing two chains formed by complementary N—H⋯O and C—H⋯O inter­actions and their association through additional C—H⋯O inter­actions.

a . DOI: 10.1107/S1600536814024039/is5380fig3.tif
Packing diagram viewed down the *a* axis showing an edge view of several ribbons.

CCDC reference: 1031959

Additional supporting information:  crystallographic information; 3D view; checkCIF report

## Figures and Tables

**Table 1:** Hydrogen-bond geometry (, )

*D*H*A*	*D*H	H*A*	*D* *A*	*D*H*A*
N5H5*A*O4	0.92	1.97	2.6208(13)	126
N5H5*A*O4^i^	0.92	2.34	3.2065(13)	158
C15H15O3^i^	0.95	2.59	3.4853(15)	158
C18H18O1^ii^	0.95	2.40	3.1887(16)	140

Symmetry codes: (i) 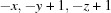; (ii) 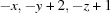.

## supplementary crystallographic information

### S1. Comment

Functionalized pyrazoles have received much attention from chemists in recent decades due to their wide use as building blocks of various pyrazole-containing structures such as pyrazoloisoquinolines (Abramov *et al*., 2001), pyrazolopyrimidines (Quiroga *et al.*, 2001), pyrazolopyridines (Wu *et al.*, 2006), pyrazolopyrazines (El-Emary, 2006) and pyrazolotriazoles (Rangnekar & Dhamnaskar, 1988). In addition, pyrazole-containing compounds have shown outstanding biological activities such as anti-microbial (Mashevskaya *et al.*, 2001), anti-viral (Janus *et al*., 1999), anti-tumor (Park *et al.*, 2005; Bouabdallah *et al*., 2006), anti-histaminic (Yıldırım *et al.*, 2005) and anti-depressant (Bailey *et al.*, 1985) applications as well as in insecticides and fungicides (Chu & Cutler, 1986). Some azopyrazole derivatives have many applications in dyes (Karci & Demircah, 2006; Vicentini *et al.*, 1998). In light of these factors, we report here the synthesis and crystal structure determination of the title compound.

The title molecule exists in an "extended" conformation aided by the intramolecular N5—H5A···O4 interaction which is half of the bifurcated hydrogen bonding involving H5a (Fig. 1 and Table 1). The rings C1–C6, N3/C11–C14 and C16–C21, respectively, make dihedral angles of 29.17 (6), 65.47 (4) and 9.91 (7)° with the central N1/N2/C7/C8/C9 ring. In the crystal, complementary N5—H5A···O4^i^ and C15—H15···O3^i^ (i: -*x*, 1 - *y*, 1 - *z*) interactions form dimers which are further associated into ribbons running parallel to the *b* axis through complementary C18—H18···O1^ii^ (ii: -*x*, 2 - *y*, 1 - *z*) interactions (Table 1 and Figs. 2 and 3).

### S2. Experimental

In 20 ml of ethanol, a mixture of 5.06 g m (0.02 mol) of 3-methyl-1-phenyl-5-(1*H*-pyrrol-1-yl)-4,5-dihydro-1*H*-pyrazole-4-carbaldehyde and 3.96 g m (0.02 mol) of (2,4-dinitrophenyl)hydrazine was heated under reflux for 8 h. The resulting solid product was filtered off, dried under vacuum and crystallized from dioxane to furnish red-orange crystals in a sufficient quality for X-ray diffraction. *M*.p 491–493 K, yield 71%.

### S3. Refinement

H-atoms attached to carbon were placed in calculated positions (C—H = 0.95–0.98 Å) while that attached to nitrogen was placed in a location derived from a difference map and its parameters adjusted to give N—H = 0.92 Å. All were included as riding contributions with isotropic displacement parameters 1.2 - 1.5 times those of the attached atoms.

### Crystal data

**Table:**

C_21_H_17_N_7_O_4_	*F*(000) = 896
*M**_r_* = 431.42	*D*_x_ = 1.463 Mg m^−^^3^
Monoclinic, *P*2_1_/*n*	Cu *K*α radiation, λ = 1.54178 Å
*a* = 5.7955 (1) Å	Cell parameters from 9846 reflections
*b* = 15.4472 (4) Å	θ = 6.3–72.1°
*c* = 21.9289 (5) Å	µ = 0.88 mm^−^^1^
β = 93.831 (1)°	*T* = 150 K
*V* = 1958.78 (8) Å^3^	Column, red-orange
*Z* = 4	0.17 × 0.10 × 0.09 mm

### Data collection

**Table:**

Bruker D8 VENTURE PHOTON 100 CMOS diffractometer	3854 independent reflections
Radiation source: INCOATEC IµS micro–focus source	3425 reflections with *I* > 2σ(*I*)
Mirror monochromator	*R*_int_ = 0.034
Detector resolution: 10.4167 pixels mm^-1^	θ_max_ = 72.1°, θ_min_ = 3.5°
ω scans	*h* = −7→6
Absorption correction: numerical (*SADABS*; Bruker, 2014)	*k* = −19→19
*T*_min_ = 0.89, *T*_max_ = 0.93	*l* = −26→26
30722 measured reflections	

### Refinement

**Table:**

Refinement on *F*^2^	Primary atom site location: structure-invariant direct methods
Least-squares matrix: full	Secondary atom site location: difference Fourier map
*R*[*F*^2^ > 2σ(*F*^2^)] = 0.033	Hydrogen site location: mixed
*wR*(*F*^2^) = 0.089	H-atom parameters constrained
*S* = 1.03	*w* = 1/[σ^2^(*F*_o_^2^) + (0.0466*P*)^2^ + 0.6128*P*] where *P* = (*F*_o_^2^ + 2*F*_c_^2^)/3
3854 reflections	(Δ/σ)_max_ < 0.001
290 parameters	Δρ_max_ = 0.20 e Å^−^^3^
0 restraints	Δρ_min_ = −0.27 e Å^−^^3^

### Special details

**Table:**

Geometry. All e.s.d.'s (except the e.s.d. in the dihedral angle between two l.s. planes) are estimated using the full covariance matrix. The cell e.s.d.'s are taken into account individually in the estimation of e.s.d.'s in distances, angles and torsion angles; correlations between e.s.d.'s in cell parameters are only used when they are defined by crystal symmetry. An approximate (isotropic) treatment of cell e.s.d.'s is used for estimating e.s.d.'s involving l.s. planes.
Refinement. Refinement of *F*^2^ against ALL reflections. The weighted *R*-factor *wR* and goodness of fit *S* are based on *F*^2^, conventional *R*-factors *R* are based on *F*, with *F* set to zero for negative *F*^2^. The threshold expression of *F*^2^ > σ(*F*^2^) is used only for calculating *R*-factors(gt) *etc*. and is not relevant to the choice of reflections for refinement. *R*-factors based on *F*^2^ are statistically about twice as large as those based on *F*, and *R*- factors based on ALL data will be even larger. H-atoms attached to carbon were placed in calculated positions (C—H = 0.95 - 0.98 Å) while that attached to nitrogen was placed in a location derived from a difference map and its parameters adjusted to give N—H = 0.91 Å. All were included as riding contributions with isotropic displacement parameters 1.2 - 1.5 times those of the attached atoms.

### Fractional atomic coordinates and isotropic or equivalent isotropic displacement parameters (Å^2^)

**Table:**

	*x*	*y*	*z*	*U*_iso_*/*U*_eq_	
O1	−0.33378 (17)	0.99482 (6)	0.45404 (5)	0.0375 (2)	
O2	−0.58503 (18)	0.90272 (7)	0.41597 (5)	0.0501 (3)	
O3	−0.49349 (16)	0.60444 (6)	0.45127 (4)	0.0358 (2)	
O4	−0.18232 (15)	0.54965 (6)	0.49537 (4)	0.0332 (2)	
N1	0.97263 (17)	0.60556 (6)	0.70725 (5)	0.0242 (2)	
N2	0.94167 (18)	0.69340 (6)	0.70316 (5)	0.0269 (2)	
N3	0.80335 (16)	0.47264 (6)	0.66558 (4)	0.0232 (2)	
N4	0.33730 (17)	0.67100 (7)	0.58210 (5)	0.0256 (2)	
N5	0.14465 (17)	0.64891 (6)	0.54543 (5)	0.0258 (2)	
H5A	0.1113	0.5925	0.5350	0.031*	
N6	−0.40360 (19)	0.92045 (7)	0.44522 (5)	0.0327 (3)	
N7	−0.30076 (17)	0.61280 (7)	0.47743 (5)	0.0260 (2)	
C1	1.14907 (19)	0.57328 (8)	0.75003 (5)	0.0241 (2)	
C2	1.3424 (2)	0.62502 (8)	0.76363 (6)	0.0276 (3)	
H2	1.3569	0.6794	0.7440	0.033*	
C3	1.5137 (2)	0.59637 (8)	0.80620 (6)	0.0304 (3)	
H3	1.6455	0.6315	0.8159	0.036*	
C4	1.4935 (2)	0.51679 (9)	0.83452 (6)	0.0312 (3)	
H4	1.6119	0.4971	0.8632	0.037*	
C5	1.3000 (2)	0.46609 (9)	0.82076 (6)	0.0312 (3)	
H5	1.2866	0.4115	0.8402	0.037*	
C6	1.1250 (2)	0.49398 (8)	0.77893 (6)	0.0276 (3)	
H6	0.9912	0.4594	0.7702	0.033*	
C7	0.81077 (19)	0.56305 (8)	0.67051 (5)	0.0226 (2)	
C8	0.6671 (2)	0.62469 (7)	0.64242 (5)	0.0230 (2)	
C9	0.7576 (2)	0.70498 (8)	0.66482 (5)	0.0249 (2)	
C10	0.6691 (2)	0.79431 (8)	0.65187 (6)	0.0316 (3)	
H10A	0.7726	0.8365	0.6729	0.047*	
H10B	0.5136	0.8000	0.6664	0.047*	
H10C	0.6630	0.8050	0.6077	0.047*	
C11	0.9756 (2)	0.42083 (8)	0.64524 (6)	0.0304 (3)	
H11	1.1119	0.4404	0.6277	0.036*	
C12	0.9170 (3)	0.33692 (9)	0.65452 (6)	0.0365 (3)	
H12	1.0042	0.2874	0.6445	0.044*	
C13	0.7022 (2)	0.33657 (9)	0.68190 (6)	0.0348 (3)	
H13	0.6194	0.2868	0.6936	0.042*	
C14	0.6360 (2)	0.42042 (8)	0.68838 (6)	0.0284 (3)	
H14	0.4988	0.4396	0.7056	0.034*	
C15	0.4654 (2)	0.60777 (8)	0.60170 (5)	0.0236 (2)	
H15	0.4279	0.5502	0.5895	0.028*	
C16	0.0083 (2)	0.71292 (8)	0.52114 (5)	0.0247 (2)	
C17	0.0773 (2)	0.80070 (8)	0.52864 (6)	0.0304 (3)	
H17	0.2187	0.8136	0.5512	0.037*	
C18	−0.0544 (2)	0.86720 (8)	0.50421 (6)	0.0320 (3)	
H18	−0.0038	0.9254	0.5096	0.038*	
C19	−0.2638 (2)	0.84940 (8)	0.47125 (6)	0.0285 (3)	
C20	−0.3414 (2)	0.76630 (8)	0.46297 (5)	0.0269 (3)	
H20	−0.4847	0.7551	0.4408	0.032*	
C21	−0.2072 (2)	0.69842 (7)	0.48756 (5)	0.0241 (2)	

### Atomic displacement parameters (Å^2^)

**Table:**

	*U*^11^	*U*^22^	*U*^33^	*U*^12^	*U*^13^	*U*^23^
O1	0.0399 (5)	0.0267 (5)	0.0456 (6)	0.0029 (4)	0.0011 (4)	0.0057 (4)
O2	0.0423 (6)	0.0412 (6)	0.0629 (7)	0.0049 (5)	−0.0245 (5)	0.0074 (5)
O3	0.0296 (5)	0.0349 (5)	0.0410 (5)	−0.0028 (4)	−0.0129 (4)	−0.0048 (4)
O4	0.0302 (5)	0.0241 (5)	0.0442 (5)	0.0018 (4)	−0.0058 (4)	−0.0011 (4)
N1	0.0234 (5)	0.0212 (5)	0.0272 (5)	0.0005 (4)	−0.0034 (4)	−0.0015 (4)
N2	0.0285 (5)	0.0212 (5)	0.0305 (5)	0.0007 (4)	−0.0028 (4)	−0.0014 (4)
N3	0.0242 (5)	0.0210 (5)	0.0240 (5)	0.0003 (4)	−0.0023 (4)	−0.0007 (4)
N4	0.0224 (5)	0.0259 (5)	0.0276 (5)	0.0003 (4)	−0.0039 (4)	−0.0006 (4)
N5	0.0237 (5)	0.0232 (5)	0.0297 (5)	−0.0004 (4)	−0.0052 (4)	−0.0014 (4)
N6	0.0317 (6)	0.0313 (6)	0.0343 (6)	0.0039 (5)	−0.0024 (5)	0.0050 (4)
N7	0.0252 (5)	0.0278 (5)	0.0248 (5)	0.0003 (4)	−0.0012 (4)	−0.0024 (4)
C1	0.0217 (5)	0.0265 (6)	0.0237 (6)	0.0024 (5)	−0.0016 (4)	−0.0029 (5)
C2	0.0267 (6)	0.0250 (6)	0.0306 (6)	−0.0015 (5)	−0.0011 (5)	−0.0014 (5)
C3	0.0241 (6)	0.0318 (7)	0.0343 (7)	−0.0025 (5)	−0.0050 (5)	−0.0052 (5)
C4	0.0273 (6)	0.0343 (7)	0.0308 (6)	0.0015 (5)	−0.0075 (5)	−0.0015 (5)
C5	0.0320 (7)	0.0303 (6)	0.0304 (6)	−0.0020 (5)	−0.0041 (5)	0.0044 (5)
C6	0.0241 (6)	0.0300 (6)	0.0282 (6)	−0.0042 (5)	−0.0024 (5)	0.0011 (5)
C7	0.0219 (5)	0.0229 (6)	0.0228 (6)	−0.0005 (4)	−0.0006 (4)	−0.0016 (4)
C8	0.0232 (6)	0.0229 (6)	0.0229 (6)	0.0006 (4)	0.0008 (4)	−0.0002 (4)
C9	0.0251 (6)	0.0240 (6)	0.0255 (6)	0.0004 (5)	0.0003 (4)	−0.0010 (4)
C10	0.0351 (7)	0.0226 (6)	0.0359 (7)	0.0012 (5)	−0.0053 (5)	−0.0012 (5)
C11	0.0312 (6)	0.0287 (6)	0.0317 (6)	0.0048 (5)	0.0047 (5)	−0.0013 (5)
C12	0.0490 (8)	0.0243 (6)	0.0362 (7)	0.0084 (6)	0.0033 (6)	−0.0006 (5)
C13	0.0465 (8)	0.0247 (6)	0.0329 (7)	−0.0059 (6)	−0.0009 (6)	0.0029 (5)
C14	0.0271 (6)	0.0295 (6)	0.0281 (6)	−0.0031 (5)	−0.0009 (5)	0.0007 (5)
C15	0.0244 (6)	0.0215 (6)	0.0246 (6)	−0.0010 (4)	−0.0001 (4)	−0.0004 (4)
C16	0.0232 (6)	0.0257 (6)	0.0251 (6)	0.0015 (5)	0.0002 (4)	0.0011 (4)
C17	0.0264 (6)	0.0268 (6)	0.0371 (7)	−0.0019 (5)	−0.0059 (5)	0.0011 (5)
C18	0.0306 (7)	0.0250 (6)	0.0396 (7)	−0.0020 (5)	−0.0033 (5)	0.0027 (5)
C19	0.0273 (6)	0.0280 (6)	0.0299 (6)	0.0040 (5)	−0.0014 (5)	0.0045 (5)
C20	0.0243 (6)	0.0310 (6)	0.0251 (6)	0.0019 (5)	−0.0018 (4)	0.0001 (5)
C21	0.0241 (6)	0.0244 (6)	0.0235 (6)	−0.0011 (5)	0.0002 (4)	−0.0012 (4)

### Geometric parameters (Å, º)

**Table:**

O1—N6	1.2291 (15)	C5—H5	0.9500
O2—N6	1.2255 (15)	C6—H6	0.9500
O3—N7	1.2279 (13)	C7—C8	1.3822 (16)
O4—N7	1.2416 (13)	C8—C9	1.4213 (16)
N1—C7	1.3636 (15)	C8—C15	1.4469 (16)
N1—N2	1.3708 (14)	C9—C10	1.4928 (16)
N1—C1	1.4305 (14)	C10—H10A	0.9800
N2—C9	1.3254 (15)	C10—H10B	0.9800
N3—C11	1.3769 (16)	C10—H10C	0.9800
N3—C14	1.3806 (16)	C11—C12	1.3589 (19)
N3—C7	1.4012 (15)	C11—H11	0.9500
N4—C15	1.2834 (16)	C12—C13	1.418 (2)
N4—N5	1.3747 (14)	C12—H12	0.9500
N5—C16	1.3527 (15)	C13—C14	1.3609 (19)
N5—H5A	0.9184	C13—H13	0.9500
N6—C19	1.4579 (16)	C14—H14	0.9500
N7—C21	1.4410 (15)	C15—H15	0.9500
C1—C6	1.3906 (17)	C16—C17	1.4201 (17)
C1—C2	1.3921 (17)	C16—C21	1.4246 (16)
C2—C3	1.3891 (17)	C17—C18	1.3677 (18)
C2—H2	0.9500	C17—H17	0.9500
C3—C4	1.3858 (19)	C18—C19	1.3973 (18)
C3—H3	0.9500	C18—H18	0.9500
C4—C5	1.3847 (18)	C19—C20	1.3683 (18)
C4—H4	0.9500	C20—C21	1.3924 (17)
C5—C6	1.3898 (17)	C20—H20	0.9500
			
C7—N1—N2	110.87 (9)	N2—C9—C10	119.82 (11)
C7—N1—C1	130.77 (10)	C8—C9—C10	128.81 (11)
N2—N1—C1	118.20 (9)	C9—C10—H10A	109.5
C9—N2—N1	105.75 (9)	C9—C10—H10B	109.5
C11—N3—C14	108.66 (10)	H10A—C10—H10B	109.5
C11—N3—C7	125.81 (10)	C9—C10—H10C	109.5
C14—N3—C7	124.97 (10)	H10A—C10—H10C	109.5
C15—N4—N5	115.85 (10)	H10B—C10—H10C	109.5
C16—N5—N4	118.63 (10)	C12—C11—N3	108.17 (12)
C16—N5—H5A	119.2	C12—C11—H11	125.9
N4—N5—H5A	122.1	N3—C11—H11	125.9
O2—N6—O1	123.59 (11)	C11—C12—C13	107.61 (12)
O2—N6—C19	118.15 (11)	C11—C12—H12	126.2
O1—N6—C19	118.26 (11)	C13—C12—H12	126.2
O3—N7—O4	122.13 (10)	C14—C13—C12	107.58 (12)
O3—N7—C21	119.32 (10)	C14—C13—H13	126.2
O4—N7—C21	118.55 (9)	C12—C13—H13	126.2
C6—C1—C2	120.80 (11)	C13—C14—N3	107.98 (11)
C6—C1—N1	121.12 (11)	C13—C14—H14	126.0
C2—C1—N1	118.04 (11)	N3—C14—H14	126.0
C3—C2—C1	119.34 (12)	N4—C15—C8	119.65 (11)
C3—C2—H2	120.3	N4—C15—H15	120.2
C1—C2—H2	120.3	C8—C15—H15	120.2
C4—C3—C2	120.38 (12)	N5—C16—C17	119.99 (11)
C4—C3—H3	119.8	N5—C16—C21	123.88 (11)
C2—C3—H3	119.8	C17—C16—C21	116.13 (11)
C5—C4—C3	119.70 (12)	C18—C17—C16	121.76 (12)
C5—C4—H4	120.1	C18—C17—H17	119.1
C3—C4—H4	120.1	C16—C17—H17	119.1
C4—C5—C6	120.88 (12)	C17—C18—C19	119.82 (12)
C4—C5—H5	119.6	C17—C18—H18	120.1
C6—C5—H5	119.6	C19—C18—H18	120.1
C5—C6—C1	118.87 (11)	C20—C19—C18	121.32 (11)
C5—C6—H6	120.6	C20—C19—N6	119.01 (11)
C1—C6—H6	120.6	C18—C19—N6	119.67 (11)
N1—C7—C8	107.55 (10)	C19—C20—C21	118.99 (11)
N1—C7—N3	122.82 (10)	C19—C20—H20	120.5
C8—C7—N3	129.63 (10)	C21—C20—H20	120.5
C7—C8—C9	104.47 (10)	C20—C21—C16	121.97 (11)
C7—C8—C15	126.04 (11)	C20—C21—N7	115.90 (10)
C9—C8—C15	129.44 (11)	C16—C21—N7	122.13 (10)
N2—C9—C8	111.35 (10)		
			
C7—N1—N2—C9	1.25 (13)	C14—N3—C11—C12	0.56 (14)
C1—N1—N2—C9	−174.68 (10)	C7—N3—C11—C12	172.24 (11)
C15—N4—N5—C16	−177.12 (11)	N3—C11—C12—C13	−0.41 (15)
C7—N1—C1—C6	−26.82 (18)	C11—C12—C13—C14	0.11 (16)
N2—N1—C1—C6	148.16 (11)	C12—C13—C14—N3	0.22 (15)
C7—N1—C1—C2	155.46 (12)	C11—N3—C14—C13	−0.48 (14)
N2—N1—C1—C2	−29.57 (15)	C7—N3—C14—C13	−172.26 (11)
C6—C1—C2—C3	0.61 (18)	N5—N4—C15—C8	−177.73 (10)
N1—C1—C2—C3	178.34 (11)	C7—C8—C15—N4	175.05 (11)
C1—C2—C3—C4	0.50 (19)	C9—C8—C15—N4	−1.91 (19)
C2—C3—C4—C5	−0.8 (2)	N4—N5—C16—C17	6.28 (17)
C3—C4—C5—C6	−0.1 (2)	N4—N5—C16—C21	−173.53 (11)
C4—C5—C6—C1	1.2 (2)	N5—C16—C17—C18	179.26 (12)
C2—C1—C6—C5	−1.45 (19)	C21—C16—C17—C18	−0.92 (19)
N1—C1—C6—C5	−179.11 (11)	C16—C17—C18—C19	0.5 (2)
N2—N1—C7—C8	−1.12 (13)	C17—C18—C19—C20	0.3 (2)
C1—N1—C7—C8	174.14 (11)	C17—C18—C19—N6	−179.94 (12)
N2—N1—C7—N3	179.54 (10)	O2—N6—C19—C20	−1.03 (18)
C1—N1—C7—N3	−5.19 (19)	O1—N6—C19—C20	178.62 (12)
C11—N3—C7—N1	−60.95 (16)	O2—N6—C19—C18	179.19 (13)
C14—N3—C7—N1	109.43 (13)	O1—N6—C19—C18	−1.16 (18)
C11—N3—C7—C8	119.87 (14)	C18—C19—C20—C21	−0.56 (19)
C14—N3—C7—C8	−69.75 (17)	N6—C19—C20—C21	179.67 (11)
N1—C7—C8—C9	0.52 (12)	C19—C20—C21—C16	0.08 (18)
N3—C7—C8—C9	179.80 (11)	C19—C20—C21—N7	179.52 (11)
N1—C7—C8—C15	−177.05 (11)	N5—C16—C21—C20	−179.55 (11)
N3—C7—C8—C15	2.2 (2)	C17—C16—C21—C20	0.64 (17)
N1—N2—C9—C8	−0.90 (13)	N5—C16—C21—N7	1.04 (18)
N1—N2—C9—C10	177.49 (11)	C17—C16—C21—N7	−178.78 (11)
C7—C8—C9—N2	0.25 (13)	O3—N7—C21—C20	−3.71 (16)
C15—C8—C9—N2	177.71 (11)	O4—N7—C21—C20	176.00 (11)
C7—C8—C9—C10	−177.97 (12)	O3—N7—C21—C16	175.74 (11)
C15—C8—C9—C10	−0.5 (2)	O4—N7—C21—C16	−4.56 (16)

### Hydrogen-bond geometry (Å, º)

**Table:**

*D*—H···*A*	*D*—H	H···*A*	*D*···*A*	*D*—H···*A*
N5—H5*A*···O4	0.92	1.97	2.6208 (13)	126
N5—H5*A*···O4^i^	0.92	2.34	3.2065 (13)	158
C15—H15···O3^i^	0.95	2.59	3.4853 (15)	158
C18—H18···O1^ii^	0.95	2.40	3.1887 (16)	140

Symmetry codes: (i) −*x*, −*y*+1, −*z*+1; (ii) −*x*, −*y*+2, −*z*+1.
