# Supplementary figures and images for: Crystal structure of (E)-4-{[2-(2,4-di­nitro­phen­yl)hydrazin-1-yl­idene]meth­yl}-3-methyl-1-phenyl-5-(1H-pyrrol-1-yl)-1H-pyrazole
Source: Acta Crystallogr Sect E Struct Rep Online. 2014 Nov 8;70(Pt 12):o1246–7. doi: 10.1107/S1600536814024039 (PMC4257438; doi:10.1107/S1600536814024039)

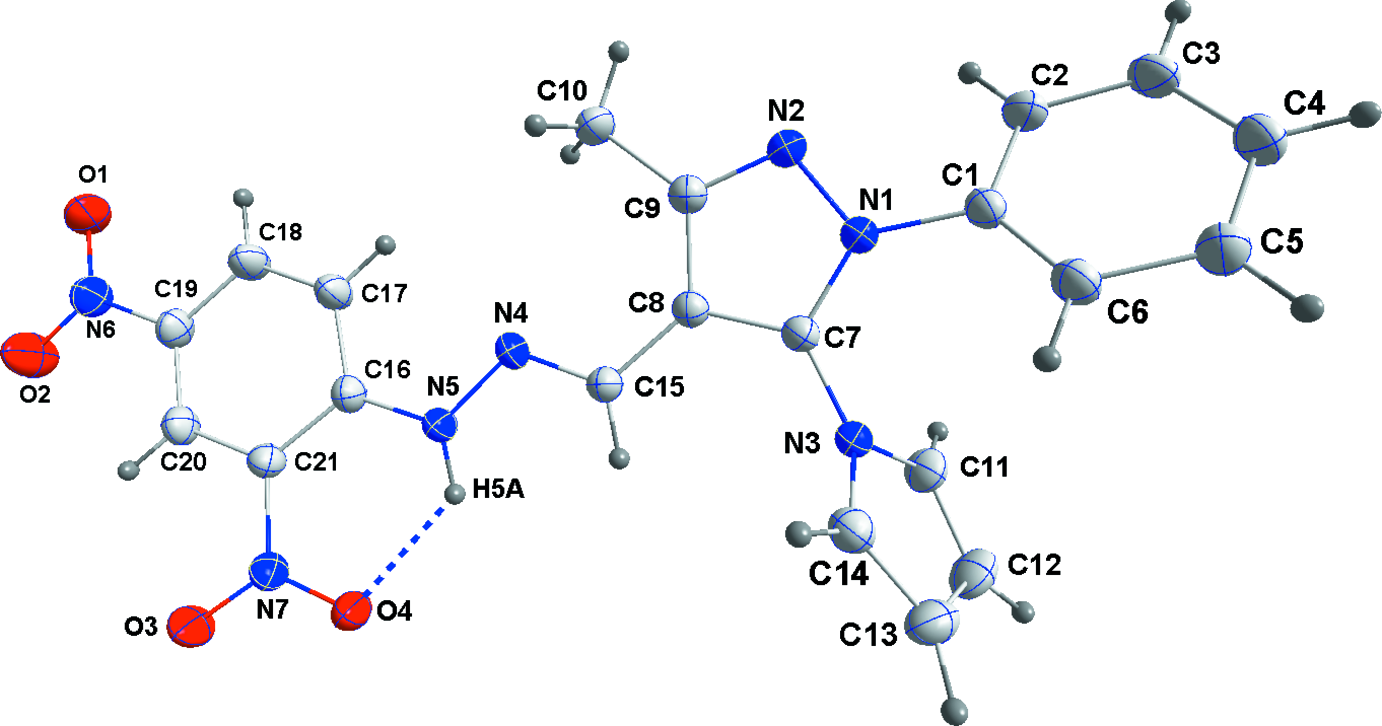

Supplement: Supplementary file 4 [file e-70-o1246-fig1.tif]

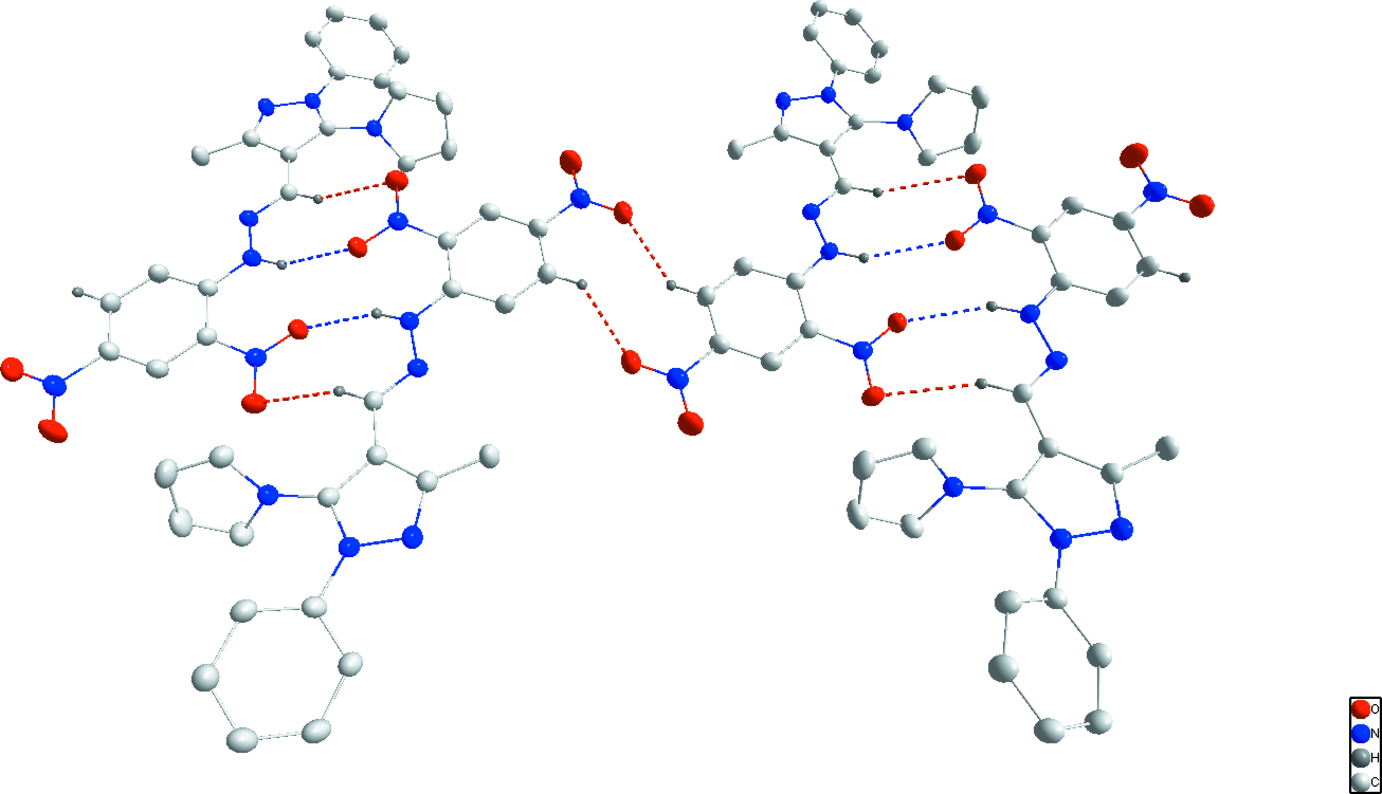

Supplement: Supplementary file 5 [file e-70-o1246-fig2.tif]

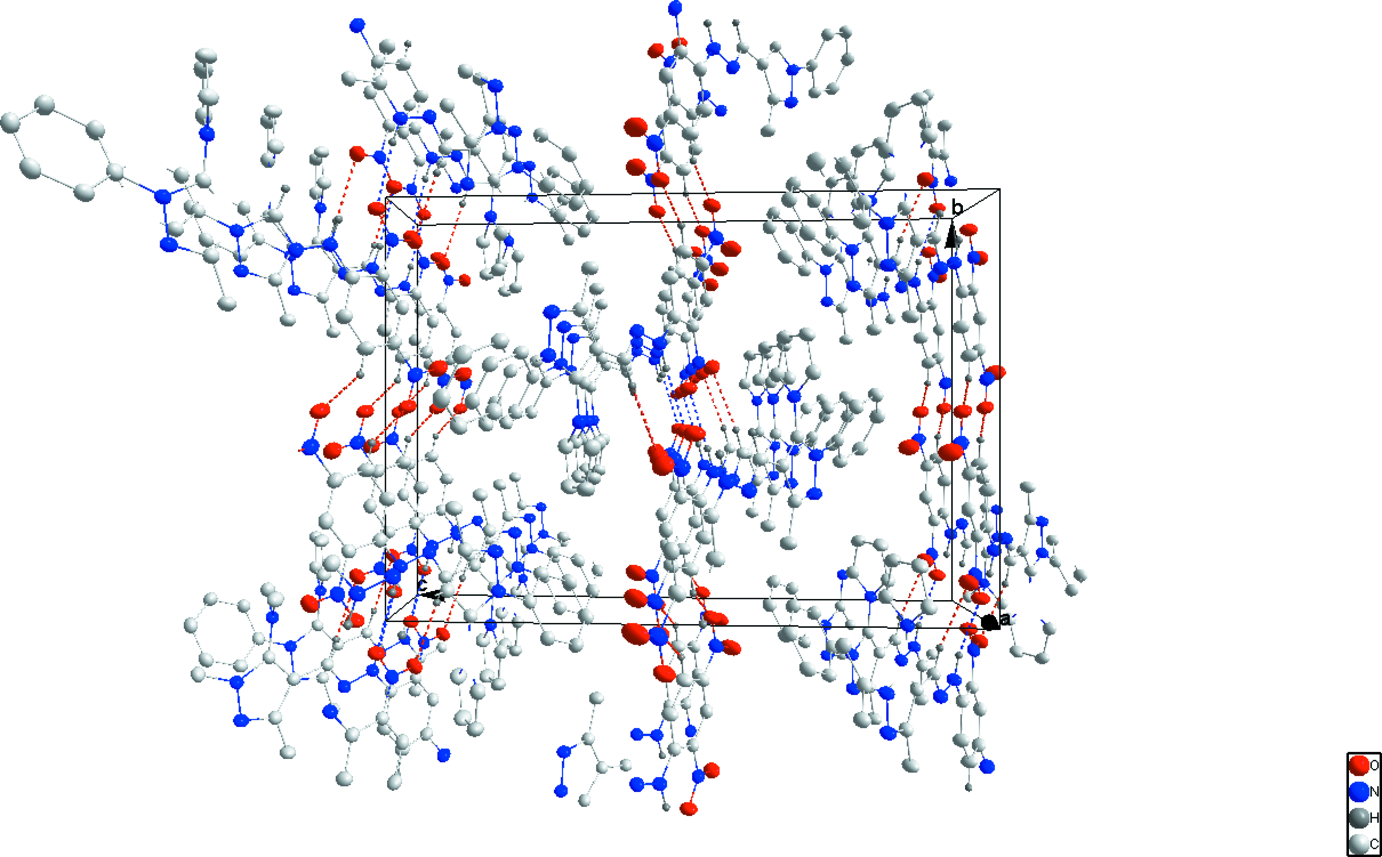

Supplement: Supplementary file 6 [file e-70-o1246-fig3.tif]
